# Supplementary material for: Molecular Characterization of Porcine Epidemic Diarrhea Virus from Field Samples in South Korea
Source: Viruses. 2023 Dec 14;15(12):2428. doi: 10.3390/v15122428 (PMC10748127; doi:10.3390/v15122428)
Supplement: Supplementary file 1 [file viruses-15-02428-s001.zip › viruses-2723508-supplementary.pdf]

Supplementary Table S1. Information of PEDV positive samples in this study.

Name	Sample	Date	Province	List of vaccines	GenBank
CNU-22S11	Fecal swab	Aug-2022	Jeonbuk	PRRSV, PRoV, PEDV	OR529200
CNU-22S16	Fecal swab	Aug-2022	Jeonbuk	PRRSV, PRoV, PEDV	OR529201
CNU-22S17	Fecal swab	Aug-2022	Jeonbuk	PRRSV, PRoV, PEDV	OR529202
CNU-22P1	Feces	Sep-2022	Chungnam	PRRSV, PRoV, PEDV	OR529203
CNU-22P2	Feces	Sep-2022	Chungnam	PRRSV, PRoV, PEDV	OR529204
CNU-22P4	Feces	Sep-2022	Chungnam	PRRSV, PRoV, PEDV	OR529205
